# Procedural determinants of fluoroscopy time in patients undergoing cardiac catheterization

**DOI:** 10.12669/pjms.35.1.294

**Published:** 2019

**Authors:** Syed Fayaz Mujtaba, Tahir Saghir, Jawaid Akbar Sial, Nadeem Hassan Rizvi

**Affiliations:** 1 Syed Fayaz Mujtaba, National Institute of Cardiovascular Diseases, Karachi, Pakistan; 2 Tahir Saghir, National Institute of Cardiovascular Diseases, Karachi, Pakistan; 3 Jawaid Akbar Sial, National Institute of Cardiovascular Diseases, Karachi, Pakistan; 4 Nadeem Hassan Rizvi National Institute of Cardiovascular Diseases, Karachi, Pakistan

**Keywords:** Angiography, Fluro time, PCI, Radiation

## Abstract

**Background & Objective:**

Due to increase in number of cardiac catheterization procedures safety concerns is an issue nowadays. Multiple diagnostic modalities use radiations, which also put a patient at higher cumulative radiation exposure. Therefore steps should be taken to minimize radiation exposure during cardiac catheterization. Hence determination of factors which prolong FT will result in better understanding of problem. This retrospective study was undertaken to determine factors responsible for prolong fluoroscopy time in patients undergoing coronary artery catheterization.

**Methods:**

This retrospective study was conducted at catheterization Laboratory National Institute of Cardiovascular Diseases, Karachi from June 2014 to June 2015. Patients of either gender, aged between 18 to 90 years undergoing cardiac catheterization procedures were included. Radiation exposure time was measured in terms of fluoroscopy time.

**Results:**

A total of 957 patients were included in this study out of which 731 were of diagnostic Coronary Angiograms (CA) and 226 were of Percutaneous Coronary Intervention (PCI). The mean age of the study participants was 54.12±10.89 years and majority 734(76.6%) were male. Mean fluoroscopy time (FT) in the patients subjected to PCI was 9.61±6.07 minutes while in cases for CA 4.17±4.13 minutes. FT for CA was observed significantly dependent on procedural access, operator’s experience, and LV angiogram. While FT for PCI was found dependent on number of stents deployed during the procedure.

**Conclusion:**

For invasive coronary angiographic procedures radial route increased fluoroscopy time. For percutaneous coronary intervention femoral and radial route fluoroscopy time were not significantly different.

## INTRODUCTION

In modern era cardiac catheterization has established its role as a diagnostic and interventional modality in cardiovascular disease.^1^-^3^ Increase in number of procedures is due to advancement in both operator expertise and improvement in hardware, hence fewer complications are associated with cardiac catheterization procedures.

Due to increased number of cardiac catheterization procedures, concerns regarding radiation safety have arisen.^4^-^6^ Increased radiation exposure has been associated with periprocedural complications including early mortality, emergent CABG, and contrast-induced nephropathy.^7^

Fluoroscopic time (FT) is most easily assessed tool for radiation exposure. Cath labs can assess their FT to make changes in their procedural techniques to minimize radiations to operator and helping staff.This is worth mentioning that cine images are not included in FT, therefore FT may not be a sole useful value for radiation dose to patient.^8^,^9^

This study aim was to find the mean fluoroscopy time of diagnostic invasive coronary angiography (ICA) and percutaneous coronary artery intervention (PCI) performed in cardiac catheterization lab of a tertiary cardiac setup. Results of this study may emphasize on reducing the fluoroscopy time to avoid excessive radiation exposure.

## METHODS

This descriptive cross sectional study was conducted at catheterization Laboratory National institute of cardiovascular diseases, Karachi from June 2014 to June 2015. It was approved by hospital ethical review committee and informed consent was taken from all patients included. Patients of both gender and age between 18 to 90 years undergoing cardiac catheterization procedures due to different indications were included. Radiation exposure time was measured in terms of fluoroscopy time (FT), minutes from time of onset of fluoroscopy till the end of procedure.

All the procedures were performed by different operators with different level of expertise and were grouped into two (consultants, senior registrar’s and post fellow trainees). Procedures were categorized into three groups depending on the nature of procedure (coronary angiography, percutaneous coronary intervention (PCI)), and two groups on basis of accesses sites (femoral and radial). Data was entered and analyzed using SPSS-21. Mean ± standard deviation (SD) and median [interquartile range (IQR)] were calculated for continuous variables such as age (years) and fluoroscopy time (FT) (minutes). Mann–Whitney U / Kruskal–Wallis test was applied to examine the differences in fluoroscopy time (FT) by baseline and procedural characteristics of the patient. Two sided p-value of ≤0.05 was be taken as criteria of statistical significance.

## RESULTS

Study sample consist of 731 patients who underwent invasive coronary angiography (ICA) and 226 patients who underwent percutaneous coronary intervention (PCI). Mean age of 731 coronary angiography patients was 53.97 ± 10.75 years, majority, 75.2% (550), were male, and access for the procedure was femoral for majority, 91.8% (671), of the patients. Mean age of 266 percutaneous coronary intervention (PCI) patients was 54.92 ± 11.67 years, 81.4% (184) were male, and procedural access was femoral in 72.1% (163) of the patients. Mean fluoroscopy time (FT) was 4.18 ± 4.13 minutes for coronary angiographic procedures and 9.61 ± 6.07 minutes for percutaneous coronary intervention (PCI) procedures.

Assessment of fluoroscopy time (FT) for coronary angiographic procedures by patient characteristics are presented in Table-I. FT was observed significantly higher in procedures with radial access, 5.25 (4.05) vs. 2.9 (3.3) minutes, p-value = <0.001, as compare to femoral access. Operator’s experience had negative association with fluoroscopy time (FT) of the procedure with p-value of <0.001. FT was higher for the procedures where LV angiogram was done, 3.3 (3.4) vs. 2.5 (3.1) minutes, p-value = 0.003. And patients with left main (LM) disease were found to have lower FT, 2.4 (2.8) vs. 3.1 (3.4) minutes, p-value = 0.020.

**Table-I:** Assessment of fluoroscopy time of invasive coronary angiographic procedures by baseline characteristics.

	Frequency (%) N=731	Fluoroscopy Time (FT)		

		Mean ± SD	Median (IQR)	P-value
*Gender*				
Male	75.2% (550)	4.19 ± 4.33 minutes	3 (3.4) minutes	0.905
Female	24.8% (181)	4.14 ± 3.45 minutes	3 (3.8) minutes
*Age*				
Up to 50 years	41.5% (303)	4.38 ± 4.87 minutes	3 (3.6) minutes	0.395
More than 50 years	58.5% (428)	4.03 ± 3.52 minutes	3 (3.4) minutes
*Accesses site*				
Femoral	91.8% (671)	4.04 ± 4.03 minutes	2.9 (3.3) minutes	<0.001*
Radial	8.2% (60)	5.7 ± 4.95 minutes	5.25 (4.05) minutes
*Operator*				
Consultant	19.3% (141)	4.24 ± 4.52 minutes	2.5 (3.5) minutes	<0.001*
Senior registrar	12.7% (93)	3.23 ± 3.26 minutes	2.3 (2.2) minutes	
Post fellow	68% (497)	4.33 ± 4.15 minutes	3.4 (3.5) minutes
LV angiogram				
Yes	60.7% (444)	4.31 ± 3.87 minutes	3.3 (3.4) minutes	0.003*
No	39.3% (287)	3.97 ± 4.5 minutes	2.5 (3.1) minutes
*Normal coronary anatomy*				
Yes	19% (139)	3.76 ± 3.25 minutes	2.7 (2.9) minutes	0.115
No	81% (592)	4.27 ± 4.31 minutes	3.1 (3.4) minutes
LM disease				
Yes	7.4% (54)	3.27 ± 2.64 minutes	2.4 (2.8) minutes	0.020*
No	92.6% (677)	4.25 ± 4.22 minutes	3.1 (3.4) minutes	

P-values are based on either Mann–Whitney U or Kruskal–Wallis test,

* Statistically significant at 5% level of significance.

Assessment of fluoroscopy time (FT) for percutaneous coronary intervention procedures by patient characteristics are presented in Table-II. No statistically significant difference in fluoroscopy time (FT) of percutaneous coronary intervention was observed by gender, age, procedural access, type of procedure, and disease anatomy. Fluoroscopy time (FT) of percutaneous coronary intervention procedure was found to be positively associated with the number of stents deployed during procedure (p-value < 0.001).

**Table-II:** Assessment of fluoroscopy time of percutaneous coronary intervention by baseline characteristics of the study sample.

	Frequency (%) N=226	Fluoroscopy Time (FT)		

		Mean ± SD	Median (IQR)	P-value
*Gender*				
Male	81.4% (184)	9.6 ± 6.03 minutes	7.8 (7.35) minutes	0.999
Female	18.6% (42)	9.65 ± 6.34 minutes	8.85 (6.8) minutes
*Age*				
Up to 50 years	37.9% (50)	9.64 ± 6.28 minutes	8 (7.4) minutes	0.859
More than 50 years	62.1% (82)	9.49 ± 5.53 minutes	8.35 (7.5) minutes
*Accesses site*				
Femoral	72.1% (163)	9.23 ± 5.85 minutes	7.9 (6.2) minutes	0.17
Radial	27.9% (63)	10.6 ± 6.56 minutes	8.4 (9) minutes
*Type of procedure*				
Elective PCI	80.5% (182)	10.03 ± 6.42 minutes	8.3 (7.5) minutes	0.089
Primary PCI	19.5% (44)	7.91 ± 4.01 minutes	6.55 (4.9) minutes
*LAD stented*				
Yes	60.2% (136)	9.97 ± 6.35 minutes	8.35 (6.7) minutes	0.273
No	39.8% (90)	9.08 ± 5.62 minutes	7.2 (7.6) minutes
*RCA stented*				
Yes	32.7% (74)	9.97 ± 6.87 minutes	8.15 (7.9) minutes	0.958
No	67.3% (152)	9.44 ± 5.66 minutes	7.9 (6.6) minutes
*LCX stented*				
Yes	16.4% (37)	11.53 ± 7.15 minutes	10.6 (9.4) minutes	0.043
No	83.6% (189)	9.24 ± 5.79 minutes	7.5 (6.2) minutes
*OM stented*				
Yes	5.8% (13)	13.2 ± 7.95 minutes	13 (9.8) minutes	0.07
No	94.2% (213)	9.39 ± 5.89 minutes	7.9 (6.6) minutes
*Number of stents placed*				
One	83.2% (188)	8.77 ± 5.2 minutes	7.4 (5.8) minutes	<0.001*
Two	13.7% (31)	12.98 ± 8.37 minutes	10.6 (11) minutes	
Three	2.7% (6)	17.9 ± 6.78 minutes	14.6 (4.5) minutes	

P-values are based on either Mann–Whitney U or Kruskal–Wallis test,

* Statistically significant at 5% level of significance.

## DISCUSSION

We found mean fluoroscopy time of 4.18 ± 4.13 minutes for invasive coronary angiographic (ICA) procedures. Our FT is almost similar to one other study i-e 4.4 min.^9^ In one study mean fluoroscopy time was 2.6 [1.7–4.5] minutes for ICA.^10^ Our fluoroscope time is much shorter than the period when ICA was in its evolution. In previous studies Fluoroscope time ranged from 5.4 to 13.5.^11^-^14^ Improvement is fluoroscopy time is attributed to both improved hardware as well as operator learning.

Increase volume of procedure in modern times is one of the major factors in understanding various anatomical anomalies and ways to negotiate them. Increased exposure to patients has resulted in better understanding of ways to deal with those natural anatomical variation and experimentation with hardware, resulting in overall short fluoroscope time.

We found a significant difference in fluoroscope time between radial vs. femoral angiographies. Same has been found in other studies.^15^,^16^ In a previous study, conducted at same center, researchers have mentioned fluoroscopy time of 6.3 ± 3.8 minutes for radial and 4.0 ± 2.9 minutes for femoral route.^15^ Whereas our study showed FT 4.04 ± 4.03 minutes for femoral and 5.7 ± 4.95 minutes for radial route. A smaller decrease in fluoroscopy time can be explained due to the fact that radial route procedures were carried out more frequently than before. Initially consultant as well as trainee doctors were reluctant for radial procedure. Increase in number of radial procedure in part was also due to patient’s preference and partly due to increased confidence of operator. Our Radial ICA time is compatible with other local centers to be advised as safe alternate method for coronary angiography.^17^ Radial procedure has a learning curve.^18^,^19^ Prolong fluoroscopy time can be attributed to the fact that procedures were done by most senior consultants as well as post fellows with relatively little experience. For the beginners radial procedure is difficult at various stages from access site puncture to engagement. Prolong fluoroscopy may be required to go through radial tortuosity, at subclavian level or while engagement. During radial approach engagement is often suboptimal. Therefore images obtained are less clear. This necessitates either repeat images or other view. This results in prolong FT.

LM disease had less fluoroscopy due to limited shots acquired and omission of LV angiogram. On the other hand Left ventricular (LV) angiogram which is presumed to prolong fluoroscopy due to change of catheter did prolong FT. LV angiogram gives us very important information regarding LV functions which can be utilized on table decision for further intervention.^20^

Our Cath lab team which performed angiographies can be divided into three groups with respect to their experience. Most senior ones were the consultant with mostly having experience > 20 years. Most junior ones, who performed majority of procedures, were post-fellowship trainees having experience of less than two years. Senior registrar was the middle group. We found that FT of the first two groups is almost comparable. While the FT of senior registrar’s was much lesser. This was surprising as consultants being most experienced group were thought to have less FT. This may be because consults are rarely performing only LHC. Most of the time these are performed either by post fellowship trainees or senior registrar, therefore their reflexes in performing LHC are not good as these assumed.

Fluoroscopy time for PCI was 9.61 ± 6.07 minutes. This is much lower than previous studies done by Federman J et al. (24 minutes) and Patee PL et al. (19 minutes) during 90’s.^21^,^22^ Unlike other local studies, we did not find any significant difference between radial and femoral route PCI.^15^,^16^

Unlike other study during the intervention two vessel stenting significantly increased fluoroscopy time.^23^ Another study involving 20,669 procedures has shown that the treatment of two or more lesions correlated with an increase in radiation exposure.^24^ Intervention requires a clear idea about size, diameter and type of stenting. Therefore each time a lesion is stented time is doubled. Therefore second stenting has more than an additive effect on fluoroscopy time.

In modern era stenting time has also reduced dramatically, in our study one stent and two stent PCI had FT 8.77 ± 5.2 minutes and 12.98 ± 8.37 minutes respectively. In 80’s for one stent and two stent fluoroscopy time was 17.1 min for single-vessel PTCA and 19.8 min for double-vessel PCI.^25^,^26^

Lesion complexity and artery involved also determines fluoroscopy time.^18^ In our study stenting of LCX and OM had longer fluoroscopy time than interventions involving other arteries. Other studies too have shown that LCX intervention took longer radiation dosage. A study with 1,827 patients undergoing angioplasty has shown that the complexity of the lesion treated, angioplasty of the CX and number of lesions treated correlated with an increase in the radiation dose.^27^ LCX is often difficult to engage and require multiple images to get a clear idea regarding size and proper position of stent.

Primary PCI required lesser fluoroscopy time. This is because only single culprit artery is involved most of the times. Due to urgency of condition decision is promptly made and multiple views are not taken. As a policy we did only culprit lesion PCI when the patients were hemodynamicaly stable.

### Study Limitations

This historical prospective study was conducted at one single center with data collection from medical records. In future more studies are needed after controlling the co-morbidities that were present in this study in the both groups.

## CONCLUSION

For invasive coronary angiographic procedures radial route increased fluoroscopy time. For percutaneous coronary intervention femoral and radial route fluoroscopy time were not significantly different.
